# In the uncharted water: Meaning-making capacity and identity negotiation of Chinese lesbian and bisexual women

**DOI:** 10.3389/fpsyg.2023.1147119

**Published:** 2023-04-17

**Authors:** Yang Hang, Xiaojun Zhang

**Affiliations:** Academy of Future Education (AoFE), Xi’an Jiaotong-Liverpool University, Suzhou, China

**Keywords:** lesbian and bisexual women, Chinese, university student, identity negotiation, meaning-making capacity, ecology of environmental systems

## Abstract

Chinese lesbian and bisexual women (LBW) often face difficulties and challenges on campus due to their multiple, socially-oppressed identities. These students have to navigate through uncharted environments to make meaning of their identities. In this qualitative study, by considering four environmental systems of student life, including the student club (microsystem), the university (mesosystem), families (exosystem), and society (macrosystem), we aim to explore what identity negotiation Chinese LBW students have in them and what their meaning-making capacity influence that identity negotiation. We find students experience identity security in the microsystem, identity differentiation-inclusion or inclusion in the mesosystem, and identity unpredictability-predictability or predictability in the exosystem and macrosystem. Moreover, they employ foundational, transitional (formulaic to foundational or symphonic), or symphonic meaning-making capacity to influence their identity negotiation. Suggestions are made for the university to create an inclusive climate accommodating students with different identities.

## Introduction

Sexual minority, namely non-heterosexual, students face various challenges due to their underprivileged sexual orientation identity. They are likely to confront bias, discrimination, and aggressive behaviors from peers if the campus climate is hostile or is not designed for their protection and inclusion (Renn, 2003). In China, studies showed that 40.7% of 751 LGBTQ students reported hearing slanders (Wei and Liu, 2015), and 85% and around 40% of 732 LGBTQ students felt depressed and thought about committing suicide (Wei and Liu, 2019), respectively, due to their sexual orientation. In addition, they are susceptible to school bullying (Wei and Liu, 2015), psychological problems (Wei and Liu, 2019), and sleep problems (Wu et al., 2021).

Although the Diagnostic and Statistical Manual of Psychiatry launched by the Chinese Psychiatric Association in 2001 excluded homosexuality from the list of mental illnesses (CMA, 2001), homosexual couples still do not have the legal right to get married or adopt children in China (Hu et al., 2020). Negative attitudes and biases against the sexual minority group continue among Chinese college students (Liu et al., 2010). Inheriting the traditional Confucian culture, most Chinese people attach great importance to filial piety and the continuity of the family line. Non-heterosexual is considered immoral and unfilial (Wei and Liu, 2015, 2019). As a consequence, sexual minority students often suffer from stigma, discrimination, and stress in universities, families, and society.

When their sexual minority identity intersects with the woman identity, the pressure on Chinese sexual minority woman students is even greater because of the rigid gender role expectations. As claimed in previous research, Chinese teachers and parents often encouraged students to obey traditional gender norms. Woman students were confined to the traditional roles of serving families (Li et al., 2013; Yang and Gao, 2021) and carrying on the family line. Shi et al. (2022) verified that Chinese woman university students who were lesbian and bisexual reported a higher risk of depressive symptoms than their heterosexual peers.

### Identity negotiation in the ecology of environmental systems

Some sexual minority students may be passively impacted and suppress their sense of identity to fit into the larger community and meet others’ expectations (Abes and Jones, 2004) while others can actively respond to or bravely challenge the dominant power structures (Linder et al., 2019). The latter implies an identity negotiation process of asserting, defining, modifying, challenging, and/or supporting one’s own and others’ desired self-images (Ting-Toomey, 2005).

Identity negotiation is a mutual communication activity between individuals and the environment. It is fluid and perceived or performed in dynamic ways (Ethier and Deaux, 1994). Ting-Toomey (2005) proposed a boundary-crossing spectrum of identity negotiation spanning from more negative experiences or outcomes (e.g., identity vulnerability, differentiation, unpredictability, etc.) to more positive ones (e.g., identity security, inclusion, predictability, etc). It recognizes the dynamism of students’ experiences (Gravett et al., 2020). According to this theory, students are likely to experience identity security in a familiar environment, while identity vulnerability in an unfamiliar environment. They will “feel included when their group membership identities are endorsed” during the positive group contact, whilst “identity differentiation when their group membership identities are stigmatized in hostile out-group contact situations” (Ting-Toomey, 2005). If interacting with familiar others, students tend to trust them and experience identity predictability. In turn, they will distrust unfamiliar others and undergo identity unpredictability. Obtaining certain capacity is necessary for navigating competent identity negotiation processes (Ting-Toomey, 2005).

A wide array of previous studies shed light on sexual minority students’ identity development and negotiation within the environment of universities (Miller et al., 2021). According to Hughes and Hurtado (2018), inclusive curricular and co-curricular diversity activities, along with LGBTQIA+ student organizations, would contribute to the identity salience of LGBT students. Duran and Garcia (2021) investigated the perceptions of heterosexist and gendered norms and identity negotiation of 20 queer women of color in culturally based sororities on campus. Some of them reduced attention to their sexual and gender identities strategically, whereas others “asserted these identities to disrupt hegemonic norms” (p. 186). Nevertheless, the identity negotiation of sexual minority university students was dominantly discussed in the Western context (Brown et al., 2004; Yost and Gilmore, 2011; Garvey et al., 2017, 2019). Existing research on sexual minority university students in the Chinese context is in small quantity and prioritized quantitative methods (Wei and Liu, 2015, 2019; Huang et al., 2018; Wang et al., 2021; Wu et al., 2021). Also, they stressed the vulnerability of Chinese sexual minority students and centered on their psychological problems. In light of that, it is essential to get a more nuanced understanding of how this group of students actively negotiate their identity in the Eastern context through qualitative research.

Furthermore, the university is not an isolated island. According to the human development theory (Bronfenbrenner, 1977), numerous systems coexist within the ecological environment, both internally in the university and externally in society. They are microsystem, mesosystem, exosystem, and macrosystem (Bronfenbrenner, 1977). The microsystem is a proximal and immediate setting that contains individuals. Interrelations among microsystems create a mesosystem. The exosystem does not contain but impinges on individuals indirectly, while the macrosystem exerts the most distal influence. It is argued that progressive interaction and mutual accommodation between individuals and environments are connected (Bronfenbrenner, 1977). When cultural norms, values, and attitudes of the majority or superiority (e.g., heterosexism, masculinity) occupy the dominant space in the environment, cultural minorities or subordinate groups may either endure or grapple with their identity (Abes and Jones, 2004).

It is necessary to take these systems into consideration holistically since they interact interdependently and reciprocally with each other (Bronfenbrenner, 1977), and influence students’ perceptions and negotiation of identities. The university, whose climate has profound impacts on students (Thapa et al., 2013), constitutes the mesosystem, while the vast society embedded with traditional norms and values represents the macrosystem. There are also microsystems like student organizations (Hughes and Hurtado, 2018) and exosystems like families, local authorities, and communities. Renn (2003) framed the study on the mixed racial identities of 38 college students through the lens of developmental ecology. However, the ecology model was still confined to a campus environment. The identity negotiation of sexual minority students in different systems was rarely examined. Moreover, their agency and active role in the identity negotiation process lacked investigation.

### Meaning-making capacity

Some research shed light on sexual minority students’ agency in their identity negotiation. Salvati and Chiorri (2023) verified that LBW’s mindfulness, a skill to attend to the inner experience in a non-judgmental manner, led to their less internalized sexual stigma. Focused on a group of LGBT student leaders and queer activists, Renn (2007) discovered an involvement-identity cycle in which increased leadership contributed to the public LGBT identity salience. Linder et al. (2019) also examined the efforts of sexual minority student activists to combat inequities on campus.

The concept of meaning-making capacity, which implies assumptions that determine one’s perceptions and organizations of life experiences (Kegan, 1994), can deepen the understanding of students’ agency in a development process. Kegan (1982, 1994) conceptualized the constructivist-developmental theory that studied the transformation of individuals in meaning-making. It describes the interrelationship among cognitive, intrapersonal, and interpersonal domains of individual development from simple to complex (Baxter Magolda, 2004). Moreover, it consists of five orders of consciousness, representing the meaning-making capacity with increasing sophistication. They associate the complex negotiation of identity dimensions with domains of development (Abes and Jones, 2004).

Based on that, Abes and Jones (2004) generalized three types of meaning-making capacity: formulaic, transitional (from formulaic to foundational), and foundational. They demonstrate an increasingly complex and stronger filter between contextual influences and lesbian college students’ perceptions and constructions of their sexual orientation and other identities. In the first category, students tend to be more passively shaped by contextual influences, while in the last category, students define their identity actively with strong self-authorship (Abes et al., 2007). Self-authorship indicates the ability to “coordinate, integrate, act upon, or invent values, beliefs, convictions, generalizations, ideas, abstractions, interpersonal loyalties, and interpersonal states” (Kegan, 1994, p. 185). Students with the transitional meaning-making capacity filter external influences on identity inconsistently.

During the identity negotiation process, the meaning-making capacity determines whether the healthy identities of Chinese woman students and sexual minorities can be developed (Abes and Jones, 2004; Abes et al., 2007). In the study carried out by Zheng (2020), Chinese woman students challenged Chinese gender norms, actively got themselves involved in global feminism, and built feminist identities. Yang (2019) investigated how Chinese sexual minorities attempted to increase their identity visibility and fight for equal rights through social media. The author also illustrated the case of a Chinese lesbian college student who bravely sued the Ministry of Education of China for its maladministration of heterosexist textbooks. These students showed a foundational meaning-making capacity to not only shield but also combat inequalities and discrimination.

Considering Chinese sexual minority woman students as active participants who negotiate their identities within the interwoven environmental systems by mobilizing their meaning-making capacity can render the description of their actual situation more comprehensive and strategies of universities to support them more effective. Therefore, this study aims to examine two research questions: (1) the identity negotiation of a specific underprivileged sexual minority group – Chinese lesbian and bisexual woman (LBW) students in different environmental systems; and (2) how meaning-making capacity impacts their identity negotiation.

Given the dearth of studies on identity negotiation in environmental systems and its relationship with meaning-making capacity, we adhere to the paradigm of constructivism in the research design. Constructivism upholds subjectivist epistemology and hermeneutical methodology (Lincoln et al., 2011) and takes the position of anti-essentialism, which refutes the essentialist belief that any phenomenon or thing has a “real, true core or essence, a consistency, and a determined ability” (Buciek, 2003, p. 53).

## Method

This study adopts the constructivist qualitative method. Constructivism assumes that knowledge is an individual or collective construction and reconstruction of reality (Lincoln et al., 2011). Constructivist qualitative research adheres to the rule that theories are generated from the researcher’s subjective interpretation of the data (Lincoln and Guba, 1985). Under its guidance, we collect qualitative data and constantly implement the iterative strategy of comparing data and coding categories during data analysis.

### Data collection and tool

Before sampling and data collection began, we obtained ethical approval from the university’s Research Ethics Committee. Each interview participant received the participant information sheet and signed the consent form at the beginning of each focus group and one-on-one interview. We first used purposeful sampling to locate LBW undergraduate students on campus and then used snowball sampling to recruit more participants. Semi-structured interviews were conducted, during which we asked not only prepared questions but also open questions that emerged from the responses of interviewees (Bryman, 2016). Examples of prepared questions in this study included “What negative and/or positive encounters have you ever had on campus regarding your sexual orientation and/or gender?” “How did you deal with problems or conflicts brought by those encounters?” “Were there any changes in your understandings of your and others’ sexual orientation and/or gender after those encounters?” “What actions did or will you take to fight for equal rights for sexual minorities and/or women?”

In total, nine LBW students enrolled in different majors and years of study volunteered to participate. Table 1 shows their background information. All participants were Chinese undergraduate students and identified as either homosexual or bisexual women. Among them, five and two participated in two focus group interviews, respectively, and two had one-on-one interviews based on their personal choices and the time available. All interviews lasted for approximately one hour and were conducted in Chinese. Interview transcripts were transcribed using the iFlytek translator software and proofread. In addition to the interviews, we observed the student club activities that aimed for diversity (e.g., LGBTQIA+, gender equity, etc.) and their WeChat groups as non-participants, which helped triangulate the interview data.

**Table 1:** Information of student interviewees.

Pseudonym	Sexual orientation	Year of study (undergraduate)	Hometown (province or municipality) in China	Way of interview
D1	Lesbian	Y3	Shenzhen	One-on-one
C2	Bisexual	Y2	Shaanxi	One-on-one
S3	Lesbian	Y2	Jiangsu	Focus group 1
F4	Bisexual	Y3	Tianjin	Focus group 1
S5	Bisexual	Y2	Jiangxi	Focus group 1
Y6	Lesbian	Y2	Zhejiang	Focus group 1
Y7	Lesbian	Y2	Zhejiang	Focus group 1
S8	Lesbian	Y2	Guangdong	Focus group 2
W9	Lesbian	Y2	Sichuan	Focus group 2

### Data analysis

Following the process of thematic analysis (Braun and Clarke, 2006), in the first place, we familiarized ourselves with each interview transcript and came out with 32 (e.g., common beliefs, comrades, intimate friends in the student club, being supported and able to express at university, revealing or concealing identity in society, etc.) and 42 (e.g., being self-content of one’s identity, wanting to do something for the group, being worried about the little self and future, etc.) initial codes for two research questions, respectively. They appeared interesting to us because they contained “the most basic segment, or element, of the raw data or information that can be assessed in a meaningful way regarding the phenomenon” (Boyatzis, 1998, p. 63).

Next, we sifted the most significant or frequently emerged codes from substantive initial codes to form three (i.e., identity security in the student club, identity inclusion or differentiation-inclusion in the university, identity predictability or unpredictability-predictability in families and society) and three (i.e., foundational meaning-making capacity and identity negotiation in the microsystem, transitional (formulaic to symphonic) or symphonic meaning-making capacity and identity negotiation in the mesosystem, transitional (formulaic to foundational) or foundational meaning-making capacity and identity negotiation in the exosystem and macrosystem) sub-themes for each of the two research questions. In the end, we further saturated and refined these themes to generate two final themes: undergoing different identity negotiation processes in four environmental systems, mobilizing the meaning-making capacity to negotiate identity in four environmental systems (see Table 2).

**Table 2:** The coding scheme.

Initial codes	Sub-themes	Final themes
In the student club: common beliefs, comrades, intimate friends, sense of belonging, activities for diversity, accessing members in the same group, meaning of identity, identity being supported, meaningful things, contributions for the group.	Identity security in the student club.	*Undergoing different identity negotiation processes in four environmental systems*.
In the university: being able to express, open and free campus atmosphere, being supported by staff, being encouraged by peers, being restricted by regulations, being prejudiced by peers, high inclusiveness, group identification, biased views, equity for students with different identities.	Identity inclusion and differentiation-inclusion in the university.	
In families and society: revealing identity, concealing identity, pressure, bias, conservative attitudes of the elderly and parents, social tag, social demarcation, group identification, social acceptance, no attack, ingrained traditional cultural norms and values, identity consistency.	Identity predictability and unpredictability-predictability in families and society.	
Being conscious of one’s identity, being acceptable of different identities, being self-contented, wanting to do something for the group, making meaning of identity, enhancing ability, self-determination, altruistic motivation, self-transformation, connecting with like-minded group members, building a sense of faith, making efforts to accomplish things, understanding the group more deeply.	Foundational meaning-making capacity and identity negotiation in the microsystem.	*Mobilizing the meaning-making capacity to negotiate identity in four environmental systems*.
Reflecting on other students’ attitudes, reflecting on one’s own experience, being shocked by other students’ views, confronting queries and stigma, assisting the group, expressing opinions, getting the voice heard, clustering with in-groups, identifying with in-groups, allowing the coexistence of different voices, not caring about other students’ biases, hoping to get recognized, classifying friends according to their acceptance of identity.	Transitional (formulaic to symphonic) or symphonic meaning-making capacity and identity negotiation in the mesosystem.	
Not believing in the little self, being worried about the unknown future, being conscious of the external pressure, reflecting on others’ views, distinguishing people and cities in terms of the inclusiveness, reflecting on self and group identity, pondering social change, pondering social (in)equality, gradually promoting social acceptance, doing things for social justice, not easy to be treated as normal, disliking distinction, distinction leading to recognition, not daring to disclose identity, no need to disclose identity, adhering to oneself.	Transitional (formulaic to foundational) or foundational meaning-making capacity and identity negotiation in the exosystem and macrosystem.	

Throughout the process, we conformed to the comparative analytical method (Krueger, 2014), continuously comparing data, codes, and categories. In addition, results drawn from the interview data were reviewed by two colleagues who were familiar with but not directly involved in the research. This peer debriefing aided in probing the data analysis and enhancing its credibility (Lincoln, 2007).

## Findings

**Figure 1:**
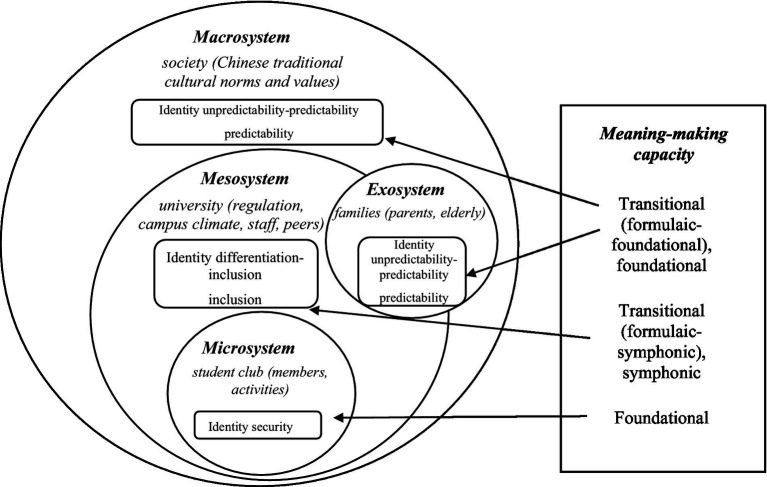
Meaning-making capacity enabled identity negotiation in the ecology of environmental systems model.

As a result, we generate two themes along with three sub-themes for each to answer two research questions based on the qualitative data. Together, they compose the model of meaning-making capacity-enabled identity negotiation in the ecology of environmental systems (shown in Figure 1).

The first theme is LBW students undergoing different identity negotiation processes in four environmental systems. First, they negotiate their identity in a secure way in the student club, which, together with its activities and members, represents the microsystem that creates a highly inclusive culture embracing diversity and voicing for the underrepresented. Second, they negotiate their identity in an inclusive or differentiating-to-inclusive way in the university. The university, including the regulation, campus climate, peers, and staff, constitutes the mesosystem with a mixed culture of inclusion and exclusion, respect, and discrimination. Third, they negotiate their identity in a predictable or unpredictable-to-predictable way in society and their families. The broader Chinese society is considered the macrosystem in favor of heterosexuality and masculinity (Hofstede et al., 2002). It keeps transmitting deeply rooted traditional norms and values to families, the impact of which becomes weaker and indirect on LBW students as they move from home to the mesosystem of the university and stay with peers in the microsystem of the student club. Thus, we consider families as the exosystem.

The second theme is LBW students mobilizing the meaning-making capacity to negotiate identity in four environmental systems. In the microsystem of the student club, LBW students form the foundational meaning-making capacity with a strong sense of self-authorship (Abes and Jones, 2004). Outside the student club, the university campus bears more uncertainties and complexities. Some LBW students construct the symphonic meaning-making capacity, that is, reconciling external influences and internal identity negotiation in harmony, while others take on a transitional (formulaic to symphonic) meaning-making capacity. Likewise, in the exosystem of families and the macrosystem of society, some LBW students transition from formulaic to foundational meaning-making capacity while others seek the foundational meaning-making capacity.

### Undergoing different identity negotiation processes in four environmental systems

LBW students experience divergent identity negotiation processes in four environmental systems, which render various affordances or constraints. The three sub-themes are: identity security in the microsystem of student club, identity inclusion or differentiation-inclusion in the mesosystem of university, identity predictability or unpredictability-predictability in the exosystem of families and macrosystem of society.

#### Identity security in the microsystem of student club

All LBW students in our interviews are members or leaders of a student club, which, together with its activities, advocates for diversity and equity. The student club, as well as its supportive members in it, form a microsystem. In general, LBW students felt their identities were secure within the space because of its familiar and friendly culture. Student Y6 said she found a sense of belonging and like-minded friends and comrades in the student club. Some LBW students became more confident and receptive to their sexual orientation and gender identities because of the close relationships with and emotional support from other club members, some of whom were even sexual majority and/or male. As one student put it:

We have the same belief. We are intimate friends and comrades. Getting along with club members, I become more self-consistent, accepting things that I did not accept before and accepting the imperfect me…Those heterosexual students join our club without much hesitation. They understand the minority “others” and treat them very well (D1).

In a word, the student club in the microsystem is a shelter for LBW students to locate in-group members or out-group “others” who join them. They accept and secure their identity as LBW students. Moreover, the space provides students with a platform to carry out activities, through which sexual minority and woman identities can be seen, and their voices for equity can be heard by others outside the student club. Student W9 praised the student club and its members for their courage. She joined in it for the sake of devoting her own power and pushing the invisible “wall” forward.

#### Identity inclusion or differentiation-inclusion in the mesosystem of university

Different from the student club, the university is replete with disparities and complexities. A lack of understanding emerges in this mesosystem with an increase in segregation and discrimination. Many LBW students mentioned experiencing biased viewpoints or unfriendly behaviors on campus. In addition, they witnessed a great number of prejudiced comments posted online by some anonymous students. Despite that, the majority of LBW students spoke highly of the university, including its inclusive and open atmosphere as well as the supportive staff. They perceived the campus climate as generally reliable and trustworthy. In addition, they felt that their identity was accepted, supported, and could be expressed. Some students claimed:

To my surprise, some university staff see our posts and ask us what assistance we need from the university side… In this year’s student club conference, it was great to see others willing to hear my and this club’s voices (D1).

It is incredible that our university allows and protects student clubs under great social pressure. I feel my identity is accepted without much pressure (C2).

However, it is inevitable that universities sometimes (enforced by the authorities) set strict regulations or impede student activities that involve sensitive topics such as sexual minorities. The student club featured in this study had to be reworded from “sexual minority” in its title to “diversity” to get the university’s approval. One student referred to this dark side:

We have many projects/activities “killed” by the university…Also, when we pull the banner in the public place of the university, we have to avoid certain sensitive words, even if these words are what we truly want to talk about (F4).

LBW students either showed a tendency to transition to or accomplish identity inclusion in this mesosystem. Most of them showed their courage and power to negotiate their identity since they mainly socialized within the university and temporarily eschewed their families and society. In other words, their struggle with the exosystem and macrosystem was usually indirect due to the intermediate spaces created by the university. LBW students maintained that they have multiple identities, which were “fluid and indefinite,” said student S8. Their sexual minority and woman identities were only parts of various identities. Currently, student identity played a more salient role as they were immersed in the campus climate and constantly interacting with staff and peers in the student club and the university. For instance, two students expressed:

These identities are just part of my life. They are not so important, at least now (C2).

Besides the sexual minority identity, I also have the student identity. My life has many sides. Lesbian is an important but not the whole part (Y6).

#### Identity predictability or unpredictability-predictability in the exosystem of families and macrosystem of society

What is more, all LBW students referred to the pressure and experience of inequality from families and society that inherited and were embedded with traditional cultural norms and values. Non-heterosexual is reckoned as shameful and immoral not only of individuals but also of their families in Chinese traditional culture, which emphasizes *chuan zong jie dai,* namely carrying on the family line. In light of that, some LBW students confessed their inclination to conceal and repress their marginalized sexual orientation identity. Facing conservative parents, student C2 dared not to disclose her bisexual orientation, “it is also unnecessary to tell them now.” Student W9 chose to conceal her homosexual identity from her mother as well because “I do not want her to worry about me.” Further, they pointed out the inferior status of women in Chinese society and the family pressure exerted on them. Some LBW students articulated:

As a woman, I find it is very hard to live in society. Things unworthy of mentioning for the male turn out to be heavy chains on us. Chinese families have traditional views of carrying on the family line (S3).

In the conservative minds of my elder relatives, women shall have a family and bear babies at an appropriate age (S8).

Their identity negotiation, hence, swayed between unpredictability and predictability. This sense of inability also emerged when LBW students confronted their parents. They were conscious of their parents’ conservative thoughts and could predict their reactions. Additionally, they introspected that their parents evaded or opposed the topic, perhaps because they were afraid of criticism from older members of the family or rumors from their colleagues in *dan wei*, namely in-system working units in China. “Face” is of great significance in Chinese culture, which means dignity.

With a salient Chinese identity, some LBW students attempted to protect their sexual orientation identity from their families and others in society, waiting for a more appropriate time in the future to voice out and remaining silent on gender inequality. Whereas, others defended their identities steadfastly and achieved a predictable identity.

### Mobilizing the meaning-making capacity to negotiate identity in four environmental systems

In correspondence to disparate identity negotiation processes in four environmental systems, LBW students mobilize different types of meaning-making capacity. It contains three sub-themes: foundational meaning-making capacity and identity negotiation in the microsystem, transitional (formulaic to symphonic) or symphonic meaning-making capacity and identity negotiation in the mesosystem, transitional (formulaic to foundational) or foundational meaning-making capacity and identity negotiation in the exosystem and macrosystem.

#### Foundational meaning-making capacity and identity negotiation in the microsystem

Almost all LBW students formed foundational meaning-making capacity in the microsystem, which secured their identity negotiation. They possessed a strong acceptance of their sexual orientation identity and self-authorship. They showed proactivity in seeking belongingness with in-group members for one thing, and contributing to affirmative action actively with the expectation of getting their voices heard by the out-group students for another. For example, several students said:

Upholding individualistic heroism, I want to exert the greatest efforts to do more things (D1).

Since I am a woman in the LGBTQ group, I want to devote myself to supporting those underprivileged identity groups with some comrades on campus (Y7).

My original intention is to do more things for the group and appeal to everyone to boycott discrimination and bullying (W9).

Additionally, certain LBW student leaders formed the leadership identity. They exhibited sensitivity to stereotypes and biases others enforced on the sexual minority and women. They also took the initiative in leading the student club and supporting its activities and demonstrated strong determination in reducing discrimination as well as appealing to equal rights. Two student club leaders stated the following:

I pursue fairness and equality. So I will make full efforts to do more and more things for the student club…I developed a sense of responsibility and changed my personality to tolerate more differences (D1).

I think we are brave, and the things we do are meaningful. I have strong personal power. I want to use my power to take a step, even if it is a small one (W9).

Their foundational meaning-making of leadership identity not only ensured their own identity security but also protected other club members’ identities and promoted connections between them.

#### Transitional (formulaic to symphonic) or symphonic meaning-making capacity and identity negotiation in the mesosystem

In the mesosystem, certain LBW students embodied the transitional meaning-making capacity (formulaic to symphonic), which made their identity negotiation undergo a transition as well, from differentiation to inclusion. They were sensitive to others’ prejudiced comments but tried to understand and reconcile the conflicts between others and themselves. For example, one student was shocked at her peers’ conservative attitudes and narrow-mindedness at university. Meanwhile, she attempted to reach a compromise with that by taking into account their divergent original living environments:

I do anticipate people in society with biased views, but I do not expect students in this university who have many years of education to be so narrow-minded. It really shocks me a lot…I think that is related to their original living environment. They come from different cities and have individual differences (D1).

Another student S5 was still striving for others’ acceptance of her bisexual identity. She confessed her sexual orientation to some intimate friends. When getting their recognition, she would be happy. Otherwise, she would distance herself from them and classify them as ordinary friends if they did not accept that. Generally speaking, she showcased a transitional meaning-making capacity (formulaic to symphonic). It made her identity negotiation linger between differentiation and inclusion.

In contrast, some LBW students showed high reflexivity and inclusivity. They contemplated that discrimination against sexual minorities and women was deeply rooted in society and not a reflection of the university. Although their abilities and impact were limited, they still had faith in bringing about changes for the sake of peaceful coexistence. This symphonic meaning-making capacity contributed to identity inclusion. For instance:

I am not angry with one’s extreme attitude. What we should do is change his or her attitude. There might be one or two out of ten people who oppose us. But there could be another nine or eight people who support us and think what we are doing is meaningful. We should not solely concentrate on those who oppose us. If they go too extreme or are hard to alter, I will ignore them (W9).

#### Transitional (formulaic to foundational) or foundational meaning-making capacity and identity negotiation in the exosystem and macrosystem

It is not easy to achieve identity predictability in the macrosystem and exosystem. A handful of LBW students were so overwhelmed by the external negative voices and attacks that they hardly employed any proactivity or inclusivity but remained sensitive and reflexive towards their sexual orientation and gender identities. They mainly underwent the transitional meaning-making stage, struggling between formulaic and foundational meaning-making capacity. Take two student as examples:

It is hard. In real life, what I can do is quite limited. We cannot conduct the Rainbow Movement. Other people do not accept that, and some even distort our actions. They bring negative effects on us. I am not willing to talk about that with others (C2).

We are like weirdos huddling together for warmth in society. We are under great pressure, discriminated against, and pointed at uncomfortably. I think in society, no matter who you are previously, you have to be forced to be “normal” in the end. But to be normal is not easy (S5).

On the contrary, several LBW students acknowledged that there were always some people in society looking at them “through tainted glasses” and coercing biased opinions upon them. Nonetheless, they believed they could gradually change others’ ideas and reduce verbal altercations through more effort. They showed a high level of sensitivity and reflexivity toward the imperfect world:

I will not impose my opinions on others. Otherwise, I will be the same as those prejudiced people. I think it is okay not to accept or understand this group, but you should not attack them. This is their life. If you can just stand by without any attack, they can feel much less pressure (D1).

What we want to do is to let others know this group is peaceful rather than aggressive. I am not saying all people in this group are good. They are as diverse as the majority in society. I just want others to treat us as normal (W9).

Moreover, they frequently reflected on self-other distinctions in identity, opinions, experiences, and attitudes resulting from disparate values, norms, and traditions. They critically thought about the social tag, change, and inclusiveness. For example, two lesbian students S8 and W9 pointed out that in big cities like Chengdu and Guangzhou, sexual minority groups were more accepted and respected than those in small cities and underdeveloped rural areas. Also, certain LBW students discussed the issue of “labeling.” Some of them expressed their dislike for the socially constructed demarcation or label, while others contemplated the positive aspect of the social label dialectically:

Being labeled is advantageous for us. It promotes the development of our group. With the label, we can be seen by others at least. People in possession of the same social label can form a strong sense of identity (Y6).

Certain LBW students also chose to boldly reveal their sexual orientation identity and argue for gender equality. Take two LBW students for instance:

I did not tell my parents. My father is stubborn. I will follow my parents’ intention to study finance first. When I earn enough money, I will contribute to the underprivileged identity groups then (S3).

I told my parents about my sexual orientation, which they strongly opposed. They said I would learn how hard this abnormal path was to take and how much discrimination I would suffer after stepping into society. But I do not care; they cannot stop me (F4).

In general, they maintained the foundational meaning-making capacity and negotiated their identities in a predictable way. They firmly adhered to their sexual minority and gender identities despite external influences.

## Discussion

This study concentrates on the identity negotiation of LBW university students, which is rarely mentioned in current literature. Plenty of previous studies paid attention to the identity negotiation of other student groups, such as international students (Kiang et al., 2020; Zhao, 2020), working-class students (Crozier et al., 2019), first-year students (Allen-Collinson and Brown, 2012; Ding and Curtis, 2020), and so on. By integrating theories of identity negotiation, meaning-making capacity, and the ecology of environmental systems holistically, it embodies LBW students’ ever-changing identity negotiation process in constant interaction with the environment (Ethier and Deaux, 1994) for one thing, and the role of their meaning-making capacity in mediating between the identity and the environment for another.

The consideration of different environmental systems contributes to the identity negotiation theory. As Ting-Toomey (2005) suggested, individuals tended to experience identity security, inclusion, or predictability in a culturally familiar or friendly environment with culturally similar others. When comparing students’ identity negotiation in the microsystem, mesosystem, exosystem, and macrosystem, we cannot completely concur with that argument. It is necessary to take into account the heterogeneity and mutability of LBW students’ identity negotiation in different environmental systems. These systems make up the ecological environment where the mainstream culture, norms, and beliefs shared among the majority and certain “small cultures” (Holliday, 1999) treasured by LBW students coexist and interplay. LBW students share a similar culture or viewpoints within the student club. They also regularly contact others who possess different values, norms, and identities in the university or families and society. Even in the same environment, some students are able to achieve identity inclusion or predictability while others are undergoing differentiation-inclusion or unpredictability-predictability transition. Besides the environmental influences, students’ agency shall be emphasized.

The meaning-making capacity manifests students’ agency in the identity negotiation process. It plays an important role in shielding LBW students’ identities from environmental influences. By adjusting the thickness and permeability of meaning-making capacity, students filter the influences of different environmental systems on their identity negotiation. They make meaning of self and others’ identities, life events, and experiences, assessing every situation and adjusting their identity negotiation depending on the environmental systems and people within them flexibly. It negates the direct impact of the environment on students, as declared in the identity negotiation theory (Ting-Toomey, 2005). Moreover, it reveals how LBW students negotiate their identities in dynamic and diverse ways. Compared with fixed components of identity negotiation competence—knowledge, mindfulness, and skills (Ting-Toomey, 2005), the fluid meaning-making capacity better delineates the lived realities of LBW students.

Besides the existent meaning-making capacity—formulaic, transitional (from formulaic to foundational), and foundational (Abes and Jones, 2004), we construct the symphonic meaning-making capacity that shows the reconciliation or balance between external influences and internal identity negotiation. There also emerges another type of transitional (formulaic to symphonic) meaning-making capacity. The concept of symphony strikes a chord with the Chinese philosophy of *he er bu tong*, namely harmony without uniformity. Some LBW students in our interviews attached importance to the coexistence of people with different sexual orientations and genders in society. Everyone is treated as normal and equal. This new finding in the Eastern context contributes to the meaning-making theory born in the Western context. Apart from the foundational meaning-making capacity that resists external influences steadfastly regardless of the environment (Abes and Jones, 2004), there is also the possibility of reconciling the internal identity and external influences harmoniously *via* the symphonic meaning-making capacity. Further, these four types of meaning-making capacity are not developed in a linear process, as implied in the meaning-making filter of Abes and Jones (2004). In face of changes in the environmental system or different environmental systems, LBW students can mobilize meaning-making capacity flexibly.

During the identity negotiation, multiple identities of LBW students emerge and interplay with their meaning-making capacity as well. With the intersection of Chinese, sexual minority, and gender identities, most LBW students retreat from proactive tendencies while sharpening their sensitivity and reflexivity. They persist in not changing or belittling their underprivileged identities but protecting and continuing them in a more explicit or implicit way. All LBW students obtain a nuanced understanding of the deeply rooted Chinese culture and tradition, such as the responsibility to carry on the family line, filial piety, seniority rules, and boy preferences, which are inherited and embodied in their family members and many others surrounding them. Furthermore, their student identity diverts their attention to more urgent and essential things, alleviating the stress brought about by other oppressed identities. However, when LBW students leave the university and step into society, their identity negotiation experiences will probably change. As predicted in the identity negotiation spectrum (Ting-Toomey, 2005), they are prone to slip into identity vulnerability, differentiation, and unpredictability. They are also likely to experience identity autonomy and change, which could be challenges or opportunities.

Last, we generate practical implications for the university. Many LBW students form a strong commitment to the student club that accommodates and cares for them. As such, more clubs and community groups like this should be cultivated on the university campus. Within these spaces, students can enhance the consciousness of their and others’ identities and actively strengthen bonds with club members to ensure identity security in this microsystem. Moreover, they can improve their self-determination and gain altruistic motivation through club activities. In this study, LBW students’ personal power and leadership identity are cultivated concomitantly.

What is more, the university shall make efforts to create a more inclusive and equal environment. On the one hand, university administrators must be willing to listen to and address underrepresented students’ concerns. In this study, the student affairs administrators play an active coordinator role and offer students valuable suggestions on adjusting their activities and avoiding sensitive words so that at least the baseline of the community could be reached. Mutual respect, along with effective communication, is demanded between administrators and students. On the other hand, university leaders may complicate their perceptions of student activism and engagement. Better promotion of mutual understanding between students with different identities, reduced bias and discrimination, and more effective approaches that equip university students with more ethnorelativist perspectives through curriculum and co-curriculum are thus called upon.

There are certain limitations of this study. Interviewees in focus groups might have experienced a stronger bias related to expressing their viewpoints in the presence of others. Considering that, we asked for their preference and got their permission before the interviews. Further, the duration of different interviews was almost the same on average despite them being individual or group ones. It was because we wanted to ensure that every interviewee had opportunities to fully express their viewpoints irrespective of the interview time. Moreover, we have yet to identify any case of formulaic meaning-making capacity. That could result from the sample size or the difficulty in locating those more inhibited and passive sexual minority students. We hope to address these issues in future research.

## Conclusion

In this qualitative study, we discover various identity negotiation experiences of Chinese LBW university students in different environmental systems. In the microsystem of the student club, they feel identity security and enjoy a sense of belonging and friendship, which are beneficial for both individual and group identities. Compared with that, the university campus as the mesosystem bears more uncertainties and complexities. Some LBW students acquire identity inclusion while others undergo differentiation-to-inclusion identity negotiation. In a similar manner, in the exosystem of families and macrosystem of society, some LBW students experience a transition from identity unpredictability to predictability. Whereas, others fully achieve identity predictability.

Furthermore, we put forward four types of meaning-making capacity: formulaic-foundational transitional, formulaic-symphonic transitional, foundational, and symphonic. They filter the impacts of four environmental systems on the identity negotiation of Chinese LBW university students. They also indicate cognitive, intrapersonal, interpersonal, and person-environmental aspects of LBW students’ agency in negotiating their identities in different environmental systems. On the one hand, this study enriches both identity negotiation and meaning-making theories. On the other hand, it has implications for the environmental systems, especially universities, to better facilitate LBW students’ identity negotiation and understand their meaning-making capacity.

## Data availability statement

The original contributions presented in the study are included in the article/supplementary material, further inquiries can be directed to the corresponding author.

## Ethics statement

The studies involving human participants were reviewed and approved by Research Ethics Committee of XJTLU. The patients/participants provided their written informed consent to participate in this study.

## Author contributions

XZ made substantial contributions to the conception of the work and revised the work critically. YH made substantial contributions to the conception, data collection and analysis of the work and drafted the work. All authors contributed to the article and approved the submitted version.

## Conflict of interest

The author declares that the research was conducted in the absence of any commercial or financial relationships that could be construed as a potential conflict of interest.

## Publisher’s note

All claims expressed in this article are solely those of the authors and do not necessarily represent those of their affiliated organizations, or those of the publisher, the editors and the reviewers. Any product that may be evaluated in this article, or claim that may be made by its manufacturer, is not guaranteed or endorsed by the publisher.

## Appendix

### Interview questions for students

1. Please give a brief introduction of yourself/yourselves, including your name, year of study, major, hometown (province or municipality), gender, and sexual orientation.
2. When were you aware of your sexual orientation? What incidents, things, or people aroused your awareness?
3. Why did you join in this student club appealing for diversity?
4. What experiences did you have in the student club?
5. Did you disclose your sexual orientation to others on campus? If no, why?
6. What negative and/or positive encounters did you have on campus regarding your sexual orientation and/or gender?
7. How did you deal with those problems or conflicts on campus?
8. Did you disclose your sexual orientation to your family members or others in society? If no, why?
9. What negative and/or positive encounters did you have in families and society regarding your sexual orientation and/or gender?
10. How did you deal with those problems or conflicts in families and society?
11. Were there any changes in your understandings of your and others’ sexual orientation and/or gender after those encounters on campus or in families and society?
12. What actions did or will you take to fight for equal rights for sexual minorities and/or women?
